# A Peer-Led Electronic Mental Health Recovery App in a Community-Based Public Mental Health Service: Pilot Trial

**DOI:** 10.2196/12550

**Published:** 2019-06-04

**Authors:** Amelia Gulliver, Michelle Banfield, Alyssa R Morse, Julia Reynolds, Sarah Miller, Connie Galati

**Affiliations:** 1 Centre for Mental Health Research Research School of Population Health The Australian National University Canberra Australia; 2 Canberra Health Services Canberra Australia

**Keywords:** peer work, computers, handheld, pilot study, mental health recovery, mental health services, community, mental disorders

## Abstract

**Background:**

There is an increasing need for peer workers (people with lived experience of mental health problems who support others) to work alongside consumers to improve recovery and outcomes. In addition, new forms of technology (tablet or mobile apps) can deliver services in an engaging and innovative way. However, there is a need to evaluate interventions in real-world settings.

**Objective:**

This exploratory proof-of-concept study aimed to determine if a peer worker–led electronic mental health (e-mental health) recovery program is a feasible, acceptable, and effective adjunct to usual care for people with moderate-to-severe mental illness.

**Methods:**

Overall, 6 consumers and 5 health service staff participated in the evaluation of a peer-led recovery app delivered at a community-based public mental health service. The peer worker and other health professional staff invited attendees at the drop-in medication clinics to participate in the trial during June to August 2017. Following the intervention period, participants were also invited by the peer worker to complete the evaluation in a separate room with the researcher. Consumers were explicitly informed that participation in the research evaluation was entirely voluntary. Consumer evaluation measures at postintervention included recovery and views on the acceptability of the program and its delivery. Interviews with staff focused on the acceptability and feasibility of the app itself and integrating a peer worker into the health care service.

**Results:**

Consumer recruitment in the research component of the study (n=6) fell substantially short of the target number of participants (n=30). However, from those who participated, both staff and consumers were highly satisfied with the peer worker and somewhat satisfied with the app. Health care staff overall believed that the addition of the peer worker was highly beneficial to both the consumers and staff.

**Conclusions:**

The preliminary findings from this proof-of-concept pilot study suggest that a peer-led program may be a feasible and acceptable method of working on recovery in this population. However, the e-mental health program did not appear feasible in this setting. In addition, recruitment was challenging in this particular group, and it is important to note that these study findings may not be generalizable. Despite this, ensuring familiarity of technology in the target population before implementing e-mental health interventions is likely to be of benefit.

## Introduction

### Background

Technology-based mental health programs continue to gather a strong evidence base [1]. These programs are rapidly increasing in uptake as both frontline health care or to complement existing mental health care services [2]. Other advantages of electronic mental health (e-mental health) programs include that they are cost-effective, scalable, and accessible [1] and can be used to empower people to maintain some control over their own care [3]. However, although these programs have been determined to be effective in trials, there are significant challenges with their implementation in routine health care, including issues with engagement and uptake [4]. Peer support interventions have been proposed as a method of increasing consumer engagement and completion of e-mental health interventions [4]. In addition, there is evidence to suggest that community samples of people with serious mental illnesses use mobile phones, mobile apps, and social media at a similar level to the general population [5,6], albeit perhaps at a marginally lower rate [7]. However, there is currently insufficient knowledge on the most effective ways of using these e-mental health tools in community mental health care settings [8] and on the role peer workers may play in these processes.

### Peer work

Peer work describes both voluntary and paid positions within consumer-operated and standard health care services [9]. Lived experience of mental illness informs the peer worker’s practices in providing support to other consumers [10,11]. Recent reviews have demonstrated that peer workers can produce a range of benefits for both the consumers, including increased independence and confidence and fostering a sense of hope [10,12], and the peer worker, including improved self-esteem and a sense of empowerment [10,13]. Peer work is rapidly gaining traction in health care internationally [6-10]. Mental health treatment landscapes are changing, and workforce shortages are placing greater demands on an already overburdened system [11]. In addition, consumer needs and priorities for treatment are evolving [11,12], particularly for recovery-focused services [10,12,13]. Thus, there is growing demand for peer workers to work alongside consumers to improve outcomes and recovery [13,14].

International evidence shows that peer work produces meaningful change for mental health consumers [10,12], but implementation of peer work programs is fragmented, and the link between programs with research evidence and current practice is poor [14]. Peer work can be challenging to implement into health care teams [15], with issues noted including a perceived lack of role clarity, issues with self-disclosure and professional boundaries, and stigma [15]. These issues can present problems for the effective integration of peer workers into the workplace and may contribute to a lack of understanding of the importance and role of peer work [13,15,16]. It has been suggested that some strategies to overcome these issues include clearly defining the peer worker’s role and the training of current staff on professional supervision and management of peer workers [15,16]. To ensure optimal delivery of peer work in the mental health system, it is vital to trial the implementation of peer recovery programs in existing health care settings.

This research examined a paid peer worker–delivered technology-based recovery intervention in a public mental health care setting.

### Aim

The aim of this study was to determine if a peer worker–led e-mental health recovery intervention was a feasible, acceptable, and effective adjunct to usual community-based treatment for people with moderate-to-severe mental illness.

## Methods

### Ethics Approval

The ethical aspects of this research were approved by the Australian Capital Territory (ACT) Health Human Research Ethics Committee (ETH.2.17.028) and the Australian National University Human Research Ethics Committee (ANU HREC 2017/338).

### Participants

The participants were 6 consumers and 5 health service staff. As per the protocol [17], because of the small sample and study location, no demographic data were collected to minimize the chance of identifying individuals. Consumer participants were people with moderate-to-severe mental illness attending a community-based public mental health service for treatment. Severity of mental illness was classified according to diagnosis, duration and intensity of symptoms, and degree of functional impairment [18]. The health professional staff participants were involved in the delivery of the program, including supervision, so they could provide valid insight into its delivery. This included 2 nurses, the peer worker’s line manager at the health service, the peer worker’s peer supervisor, and the peer worker.

### Researchers

Overall, 3 researchers involved in this study (AG, MB, and ARM) have lived experience of mental health problems and are working currently in the field as consumer researchers. The collaboration of this group with a consumer and carer advisory group offered a unique perspective on the design of the survey questions and the evaluation study overall, ensuring that both content and wording were appropriate and acceptable for the target groups [19].

### Recruitment

Participation in the e-mental health program and participation in the evaluation survey were treated separately. The peer worker and other health professional staff (ie, nurses) offered participation in the e-mental health program to attendees at the drop-in medication clinics during usual appointments at the mental health service between June and August 2017. The peer worker would also then, at a mutually convenient time, offer participation in the evaluation survey separately with the researcher. The researcher would discuss the project with consumers who would then be free to participate or decline participation in the research. The consumers were explicitly informed that participation in the evaluation was voluntary and independent of their participation in either the recovery program or the services they received at the mental health service. Recruitment fell short of the intended target of 30 consumers. Overall, of the approximately 10 people who completed the program, almost half (n=6) were willing to complete the evaluation survey.

The research team invited the health professional staff involved in the trial of the peer worker–led e-mental health program to participate in a face-to-face interview with a researcher (AG or ARM) to discuss their experiences with the delivery of the program at a mutually convenient time. A total of 4 staff interviews were conducted in December 2017, and the final interview was conducted in January 2018.

### Intervention: Peer Worker

A part-time (7 hours per week) peer worker who had completed the recognized Australian peer work qualification (Certificate IV in Mental Health Peer Work [20]) was recruited at the health service to deliver the current program. Consistent with a peer worker role, in addition to leading the e-mental health program, they were also expected to provide emotional support, develop trusted professional relationships, assist staff with recovery plans, and carry out other duties as appropriate. Key characteristics for the peer worker included (1) direct personal lived experience of using mental health services, (2) a positive experience of recovery, and (3) the ability and willingness to disclose their own personal experience of recovery to positively influence others. The peer worker was trained on the use of the e-mental health intervention, the Stay Strong app (see below), by a member of our research team (JR). In addition to their usual line management within the service, they also received professional supervision and support by an experienced peer worker supervisor.

### Stay Strong Electronic Mental Health App

The e-mental health intervention used was the Aboriginal and Islander Mental Health Initiative (Stay Strong App, which was developed by Professor Tricia Nagel through the Menzies School of Health Research and Queensland University of Technology [21,22]. It was designed for use with Aboriginal and Torres Strait Islander service users and has some culturally specific imagery and content [22]; however, it has been approved by the authors for use in non-Aboriginal and Torres Strait Islander populations.

The Stay Strong app uses a simple, highly visual design that does not require literacy or a high degree of concentration. The app assists the person to identify their own worries and strengths and helps them to establish goals for themselves in personal areas they would like to change [23]. A visual and interactive representation of the strengths (including people and relationships) and worries (weaknesses) is created using a symbolic tree. The more strengths identified, the stronger and healthier the leaves grow; conversely, the more worries identified, the more the leaves on the tree wilt and change color. The app is specifically designed to focus on recovery and can be used by workers who have some mental health training but are not necessarily health professionals [22]. The app is a structured mental health and substance use intervention designed to be used as a collaborative tool between workers and service users. To deliver the app, the peer worker acted in a coaching role and assisted the person in applying the concepts to their personal situation. The program consisted of completing the Stay Strong recovery app on Apple iPads with the support of the peer worker in 1 of 4 sessions.

### Evaluation Design

The evaluation of the current project was designed to be exploratory [17], examining the elements of the program that were useful and the barriers and facilitators to implementation in the service, and an investigation of the effectiveness of the program overall. The evaluation involved both the postintervention quantitative evaluation of a single cohort of participants and qualitative interviews with the peer worker and mental health service staff. A focus group to collect further qualitative data from participants was planned [17], but we did not proceed as no consumers from the participant pool agreed to participate. The protocol for the study has been published previously [17].

#### Evaluation Survey

The primary focus of the survey was on the acceptability of the program and its delivery for consumers. Multimedia Appendix 1 presents the evaluation survey questions. Overall, 6 questions, designed by consumer researchers in collaboration with the advisory group, assessed the acceptability of the peer worker, the e-mental health program, recovery, and self-efficacy. A seventh question addressing the participants’ rating of the group delivery was dropped from the analysis; it was not feasible to conduct the intervention in groups because of the staggered appointment and wait times for consumers. The questions asked participants to rate their agreement with each statement about the peer worker and the program on a 4-point scale (1=*No, not at all*; 2=*No, not really*; 3=*Yes, to some extent*; and 4=*Yes, definitely*). The survey also measured a single recovery outcome, which was assessed using part A of the Self-Identified Stages of Recovery (SISR) [24]. The SISR part A is a single item that describes stages of recovery and asks the participant to select the stage of recovery with which they currently identify. Participants indicate which of the 5 statements describes how they have been feeling over the past month, with higher ratings indicating more positive perceptions of recovery. The SISR has demonstrated reliability and concurrent validity [25] and convergent validity for the staged model of recovery [26]. This single item measures a unique feature of recovery not assessed by continuous measures [26].

#### Staff Interviews

Staff were interviewed about their experience with the delivery of the program within the health service. Multimedia Appendix 2 presents the interview questions for the health care staff and the peer worker. The peer worker interview focused on their experience with the delivery of the program, and the management and supervisory staff interviews focused on their observations of the feasibility of embedding a peer worker and e-mental health program within the service from an operational point of view. Interviews followed a structured protocol [17] and were recorded for accuracy.

### Analysis Strategy

Mean scores for the 6 questions assessing the acceptability of the peer worker, the e-mental health program, recovery, self-efficacy, and SISR were calculated for the participants (n=6) at postintervention. The 5 health professional interviews were conducted by AG (n=4) and ARM (n=1). Owing to the very specific areas of interest for the evaluation, and the lack of focus group data collected as per the original protocol [17], a deductive approach was taken using highly structured interview questions to target themes of interest. Notes were taken during the interviews by the researcher and were reflected to the participants after each question to ensure accuracy. Corrections to any comments were made during interviews in response to this immediate feedback. In addition, the notes were emailed to participants to provide any further comments or alterations to the data. These changes were incorporated into the notes. Participant views on themes are presented below, quotes are taken from recordings.

#### Participant Codes

Participant codes are as follows: PW=Peer Worker, PWS=Peer Worker’s Supervisor, LM=Line Manager (Health Service), HP1=Health Professional 1, and HP2=Health Professional 2.

## Results

### Participant Survey Results

Table 1 presents the results for the participant evaluation survey. There were no missing data. Participant ratings of specific aspects of the evaluation are reported below.

**Table 1:** Participant evaluation data for the peer-worker program.

Measure		Mean (SD)^a^
Self-identified stages of recovery		3.3 (1.5)
**Delivery: “Did you like:”**		
	“that a *peer worker* was assisting you with the Stay Strong program?”	3.5 (1.2)
	“completing the Stay Strong program on the *iPad (electronic device)*?”	3.2 (1.2)
	“completing the Stay Strong program during *time that you normally wait* around at the Health Service?”	3.7 (0.5)
**Program: “Did the Stay Strong program:”**		
	“give you a *sense of control over your life*?”	2.8 (1.2)
	“help you *feel that you could recover*?”	2.7 (1.0)
	“help you feel confident about your *ability to take care of yourself*?”	3.0 (1.1)

^a^The rating scale for the 6 evaluation questions was 1=No, not at all; 2= No, not really; 3=Yes, to some extent; and 4=Yes, definitely.

#### Recovery

Participant SISR scores (mean 3.33, range 2-5) indicated that, on average, participants at postintervention were between “*starting* to learn how to overcome the illness,” and being able to manage their mental illness “reasonably well.”

#### Delivery Evaluation

On average, participants were highly satisfied with (1) the peer worker assisting them with the program, (2) the iPad delivery, and (3) completing the app during their usual waiting time.

#### Program Evaluation

Participant ratings of the program overall were rated as somewhat helpful for (1) assisting them to feel that they could recover, (2) assisting them to feel a sense of control over their life, and (3) assisting them to feel confident about taking care of themselves.

### Health Service Staff Interview Results

#### Advantages of the Program

Key advantages of the program that were noted by all of the health service staff were concerning the 2 key characteristics of the program: the *peer-led* aspect of the program and the *holistic and practical approach* of the Stay Strong program. These advantages are reported below.

##### Peer Worker

The health professional staff all agreed that the main benefit of the program was the unique utility of the peer worker. This was primarily because of their ability in *normalizing* mental health problems and connecting with consumers by engaging them in a meaningful way. For example, it was noted that it was helpful for consumers to have someone they could relate to who had similar experiences and could assist with coping skills and *nonclinical* tasks such as helping them to fill out forms (eg, social security). In addition, the peer worker observed that consumers appeared to enjoy working with her, a view that was overwhelmingly supported by all health service staff. This was because of 2 key reasons: the *specific personality of the peer worker*, which was noted to be warm, empathic, and engaging, and the *validation and engagement* that people appeared to feel with having a peer worker with *authentic* lived experience working with them:

...she was able to engage with consumers like no clinician I’ve seen before.

LM

The peer worker was able to provide genuine empathy and assistance and also *validate* their experiences based on her own authentic experience, which was viewed as “...more relatable for people—more of an equal feeling” compared with when a clinician is asking the same questions [HP2]. Staff had observed participants enjoying the “...engagement and the one-on-one attention” [HP1].

##### Stay Strong Program

The program’s strengths were seen to be predominantly the holistic approach of the program and the practical skills learned. It was praised for enabling people to look “at recovery in a holistic way” [PWS], giving them “...some time to reflect on what they considered important within their life” [PW]. It was also thought to be meaningful for people because it went through their strengths, weaknesses, and supports. The app itself was seen as easy to use and was bright and engaging, and the symbolism of the tree, which was able to be printed out for people to take home, was noted as an added benefit by the peer worker.

#### Challenges of the Program

There were several key challenges noted by the staff, including *engaging people in the Stay Strong program, the iPad delivery, integrating the peer worker into the health care team*, and the *target population*. These challenges are outlined below.

##### Stay Strong Program

The Stay Strong program itself was also considered a challenge within the overall program. Although it was noted as being holistic and meaningful, it was difficult to engage people in the app in this particular group. Staff believed that people appeared uncomfortable in providing the personal information needed to engage with the app. In addition, it was noted that the app was similar to completing other routine measurement tools within the service:

Many of the people we see are so regularly asked to do things like that...it’s just another routine thing that they thought they’d have to do with us.

PW

Despite this, the peer worker thoroughly enjoyed delivering the app and found it a useful tool to bring about conversation and set meaningful goals with people.

###### Apple iPad Delivery

Overall, the view of the Apple iPad delivery was mixed but more negative than expected. All staff suggested that there was a lack of familiarity with tablet technology in this particular population. Generally, it was believed that this was up to individual preference; some enjoyed it, and others may have found the use of technology intimidating.

###### Integration of the Peer Worker Role

Overall, opinions differed on how the peer worker fit within the service across health service staff. The peer worker’s line manager believed that the integration of the peer worker role was challenging, specifically, that there was some adaptation required to integrate the peer worker into the health care team:

Orienting my health professional staff around working with a peer worker...what we noticed was being ever so mindful of [any potentially stigmatizing] language that we used.

LM

However, the 2 health service staff involved in the program delivery believed that the peer worker fit into to the team well, though noting that this may have been because of qualities unique to this specific peer worker, including her warm personality and nursing background.

###### Target Population

Staff noted that there was very low uptake of the program. The peer worker’s line manager believed that lower-than-expected levels of engagement may also have been because of the sedation of this particular population at this time: “...there’s a tendency to feel quite tired postinjection, so I do wonder if that was a barrier” [LM]. In addition, perhaps, feeling self-conscious to go off in front of other people and do something *clinical* may have also contributed to lower uptake, that perhaps there was “a social kind of anxiety, around that they don’t want to be the one to look like I’m going with the mental health clinician, outside to do a program on my mental health” [LM]. Alternatively, the line manager suggested that consumers may not have felt obliged to participate as it was a peer worker who was managing the program (as opposed to a clinician).

###### Dead Time Setting

Opinions about implementing the program within the 2-hour *dead time* after depot medication administration were mixed. The 2 health service staff working in the health service believed that for those who wanted to participate, it was an ideal time to engage this population compared with simply watching television or browsing the internet. However, the peer worker noted that there was a lot of individual difference in people’s willingness to engage with the program during that time. Instead, some preferred to spend their time sitting and relaxing, and some felt tired and wanted to use it as their “down time” [PW].

## Discussion

### Principal Findings

Overall, the study findings indicate that a peer worker–led e-mental health recovery intervention was acceptable in this single-setting evaluation study and showed some evidence of effectiveness as an adjunct to usual community-based treatment for people with moderate-to-severe mental illness. However, the program, particularly the mode of delivery (Apple iPad), did not demonstrate feasibility in this population. This study was primarily conducted as a feasibility study; thus, given the study sample was small, the results may not be generalizable to other programs, populations, or settings.

Although only a small number of potential participants engaged with the program (n=10) and the evaluation (n=6), those involved in the research reported that they liked the program. Many consumers attending the health service in this study experienced significant sedation following the administration of medication and, therefore, preferred to rest during the required wait time. Some were also unfamiliar with tablet technology; despite the ubiquity of mobile technology, especially mobile phones [27-30], the levels of familiarity and comfort with these technologies vary widely among individuals [31], and they may have been concerned about being watched or judged by others. Given that technology is increasingly being used to deliver mental health care [2], there are concerns that some people may experience digital exclusion. Service providers have been encouraged to support users to engage in services delivered electronically. In this study, the line manager noted that education for people in community-based health settings around tablet technology may be helpful for future programs using tablets.

Consistent with previous similar studies [10,12,31], health professional staff and consumers believed that the addition of the peer worker was highly beneficial. The peer worker fostered a sense of hope and provided positive role modeling for consumers, while also providing one-on-one support for tasks that were not otherwise able to be supported by health service staff. Although this is a good example of the unique elements a peer worker can add to a health care team beyond simple extra capacity, it does also raise additional considerations. As this study was part of a pilot of peer work within a public mental health service and was designed to inform role and guideline development, the scope and responsibilities of the role may have been somewhat unclear. The peer worker involved in the pilot also had nursing training; although this likely aided her integration into the health care team, it may also have blurred the boundaries of peer work versus other health care staff roles. A clear definition of the peer worker role and what they are expected to do, together with training on supervision and management of peer workers, is critical to the successful implementation of the role [15,16].

### Strengths and Limitations

The principal strength of the study was that it evaluated the feasibility and acceptability of the intervention in a real-world setting, allowing participants to self-select into the program and research evaluation in their usual treatment setting. Further strengths of the study included the consumer input into the design of the evaluation and the exploration of all perspectives, including the peer worker, consumers, and the health care staff. However, it is possible that staff may not have felt comfortable offering their honest opinions to the researchers. The study had several limitations. First, the study design only evaluated a single app, the evaluation of which cannot be generalized to other app-based programs, particularly those that may have been more suitable for this population. In addition, the study did not allow clear separation of the concept of the peer worker and the app itself. To attempt to overcome this, participants were asked to rate the aspects of the program separately (the peer worker and the app), but attribution of outcomes was still unclear. Second, given that the study only used 1 peer worker, separating out the *concept* of the peer worker from the personal characteristics of this specific peer worker was difficult. It was clear from the interviews with the staff that this individual was highly skilled, and thus, the trial may have yielded different results with different peer workers. Third, only 1 method of recruitment was applied, and it was not particularly successful in this population. It is unclear whether this was because of the population, the type of intervention offered, or the recruitment method itself. However, the use of other methods of recruitment (ie, the researchers offering participation in the program) or offering incentives would have compromised the ecological validity of this proof-of-concept trial. In addition, demographic data were not collected because of the substantial risk of the identification of participants from such a small sample. Moreover, no qualitative data from consumers could be collected via focus groups as was initially planned. Finally, the small participant sample size and lack of a control group meant that the participant findings were limited.

### Implications

This study provides a number of ways forward. First, it would be important to implement the addition of peer work or any new technology slowly and ensure adequate orientation to any new programs or changes. This includes a clear role definition for the peer worker and orientation for staff on the integration of the peer worker into the health professional team. The latter may include education on potential sensitivities and differences in language (eg, *person-first language* [32] referring to the person first and their diagnosis second) for both the peer worker and staff. Research on health professional staff attitudes toward peer work, peer workers, and any potential changes that may be reflected after the implementation of such trials would be a welcome addition to future research. Second, ensuring that a skilled and suitable peer worker is engaged in the position is vital. Careful attention to the peer worker’s level of recovery, personality, degree of autonomy, and the skills required to perform the role within a specific setting are likely help them to perform to the top of their scope and successfully integrate into the health care team. Third, future implementation studies more broadly would benefit from careful co-design. In this study, the contrast between the consumers’ and the staff’s perceptions of *dead time* was highly apparent, that is, consumers saw it as *down time*, where they could rest or watch television, whereas staff saw it this time as idle. Finally, it would also be recommended that the implementation remain adaptive, as a key part of this process is to examine the core features needed for fidelity of an intervention versus the adaptation necessary for it to work in a particular environment. We note that in this study, the group delivery as originally planned was altered to individual delivery, emphasizing the importance of remaining flexible to best accommodate the population and setting.

### Conclusions

Technology-based peer-led recovery has significant potential as an adjunct to usual treatment for people experiencing severe mental illness. The preliminary findings from this pilot trial suggest that in this setting, a peer worker was feasible and satisfactory in their role of providing the program for both health care staff and service users. However, the e-mental health program itself was not feasible in this population without modification, training, and support. The additional challenges to adoption of technology faced by this population should be borne in mind, but further large-scale research is now required to differentiate the effects of technology-based recovery work from the effects of peer work more generally. This will inform the development of a range of peer-led programs that enable peer workers to maximize their unique scope of practice.

## Appendix

Multimedia Appendix 1 Evaluation survey - participants.

Multimedia Appendix 2 Interview questions.

## Abbreviations

ACACIA: Australian Capital Territory Consumer and Carer Mental Health Research Unit
ACT: Australian Capital Territory
e-mental health: electronic mental health
SISR: Self-Identified Stages of Recovery
