# Supplementary material for: A Peer-Led Electronic Mental Health Recovery App in a Community-Based Public Mental Health Service: Pilot Trial
Source: JMIR Form Res. 2019 Jun 4;3(2):e12550. doi: 10.2196/12550 (PMC6746099; doi:10.2196/12550)
Supplement: Multimedia Appendix 1 [file formative_v3i2e12550_app1.pdf]

Multimedia Appendix 1: Evaluation survey - participants

Self-Identified Stages of Recovery

People can feel differently about life with a serious illness at different times. Below are five statements about how people may feel.

Please read **all five statements** (A-E) and answer the question that follows.

A. "I don't think people can recover from mental illness. I feel that my life is out of my control, and there is nothing I can do to help myself." ☐

B. "I have just *recently* realised that people can recover from serious mental illness. I am *just starting* to think it may be possible for me to help myself." ☐

C. "I am *starting* to learn how I can overcome the illness. I've decided I'm going to start getting on with my life." ☐

D. "I can manage the illness reasonably well now. I am doing OK, and feel fairly positive about the future." ☐

E. "I feel I am in control of my health and my life now. I am doing very well and the future looks bright." ☐

Of the five statements above, which one would you say ***most closely*** describes how you have been feeling over the **past month** about life with the illness?

Tick the box next to that statement.

The peer worker-supported Stay Strong program

We would like to ask you a few questions about what you thought about the program and how it was presented to you:

1. Did you like that a **peer worker** was assisting you with the Stay Strong program?

No, not at all	No, not really	Yes, to some extent	Yes, definitely
☐	☐	☐	☐

2. Did you like completing the Stay Strong program in a **group**?

No, not at all	No, not really	Yes, to some extent	Yes, definitely
☐	☐	☐	☐

3. Did you like completing the Stay Strong program on the **iPad (electronic device)**?

No, not at all	No, not really	Yes, to some extent	Yes, definitely
☐	☐	☐	☐

4. Did you like completing the Stay Strong program during time that you **normally wait** around at the Health Service?

No, not at all	No, not really	Yes, to some extent	Yes, definitely
☐	☐	☐	☐

5. Did your participation in the Stay Strong program give you a **sense of control over your life**?

No, not at all	No, not really	Yes, to some extent	Yes, definitely
☐	☐	☐	☐

6. Did the Stay Strong program help you **feel that you could recover**?

No, not at all	No, not really	Yes, to some extent	Yes, definitely
☐	☐	☐	☐

7. Did the Stay Strong program help you feel **confident about your ability to take care of yourself**?

No, not at all	No, not really	Yes, to some extent	Yes, definitely
☐	☐	☐	☐

8. Please add any further comments about the program?

____
____
____
____
____
____
____
____
____
____
