# Supplementary material for: A Peer-Led Electronic Mental Health Recovery App in a Community-Based Public Mental Health Service: Pilot Trial
Source: JMIR Form Res. 2019 Jun 4;3(2):e12550. doi: 10.2196/12550 (PMC6746099; doi:10.2196/12550)
Supplement: Multimedia Appendix 2 [file formative_v3i2e12550_app2.pdf]

Multimedia Appendix 2: Interview questions

1. Interview questions – health professional

Questions:

We would like to ask you a few questions about your perceptions of delivering the program at the health service:

1. Were there any observable benefits for the health service associated with the delivery of the program? If yes, what were some of the benefits you observed?

2. Were there any observable disadvantages for the health service associated with the delivery of the program? If yes, what were some of the disadvantages you observed?

3. Were there any issues with the organisation of the program or groups? If yes, please tell us more?

4. What was your experience with organising the peer worker?

5. Do you feel that participants enjoyed working with the peer worker?

6. What did you like or find useful in the delivery of the Stay Strong program?

7. What did you dislike or find unhelpful in the delivery of the Stay Strong program?

8. Were there any issues or difficulties delivering the Stay Strong program in a group setting?

9. Were there any issues or difficulties delivering the Stay Strong program using the iPads?

10. Were there any issues or difficulties delivering the Stay Strong program using “dead time” i.e., time that would participants would have just spent waiting around?

11. Did you enjoy delivering the Stay Strong program at the health service?

12. Do you have any other comments about the program?

2. Interview questions – peer worker

Questions:

We would like to ask you a few questions about your perceptions of delivering the program:

1. Do you feel that participants enjoyed working with you specifically as a peer worker? Why or why not?

2. What did you like or find useful in the delivery of the Stay Strong program?

3. What did you dislike or find unhelpful in the delivery of the Stay Strong program?

4. What did you think about delivering the Stay Strong program in a group setting? Were there any benefits or drawbacks associated with this?

5. What did you think about delivering the Stay Strong program using the iPads?

6. Were there any issues or difficulties delivering the Stay Strong program using “dead time” i.e., time that would participants would have just spent waiting around?

7. Did you enjoy delivering the Stay Strong program at the health service?

8. Do you believe that participants were satisfied with what the Stay Strong program provided?

9. Do you have any other comments about the program?
